# Interaction between arbuscular mycorrhizal fungi and dark septate endophytes in the root systems of *Populus euphratica* and *Haloxylon ammodendron* under different drought conditions in Xinjiang, China

**DOI:** 10.3389/fpls.2024.1504650

**Published:** 2025-01-27

**Authors:** Huimei Wang, Hengfang Wang, Shengtao Wei, Li Sun, Linlin Cheng

**Affiliations:** ^1^ College of Ecology Environment, Xinjiang University, Urumqi, China; ^2^ Key Laboratory of Oasis Ecology of Ministry of Education, Xinjiang University, Urumqi, China; ^3^ Xinjiang Jinghe Observation and Research Station of Temperate Desert Ecosystem, Ministry of Education, Xinjiang University, Urumqi, China

**Keywords:** arbuscular mycorrhizal fungi, dark septate endophytes, colonization strategy, colonization status, rhizosphere effect

## Abstract

**Background and Aims:**

Arbuscular mycorrhizal fungi (AMF) and dark septate endophytes (DSE) are known to enhance the tolerance of host plants to biotic and abiotic stresses, but the mechanism of their interaction under natural conditions has not been extensively studied.

**Methods:**

We analyzed the endophytic fungal diversity and colonization characteristics in the typical desert plants *Populus euphratica* and *Haloxylon ammodendron* and the relationship between them and environmental factors.

**Results:**

Except for DSE in the roots of *H. ammodendron*, the colonization rates of AMF and DSE were significantly positively correlated with drought severity. The abundance of AMF and DSE under medium and mild drought conditions was greater than that under severe drought conditions. The root colonization rate and abundance of AMF were lower than those of DSE under the same drought conditions. The species diversity and abundance of AMF and DSE in *P. euphratica* were greater than those in *H. ammodendron*. AMF were more susceptible to soil factors such as soil water content, soil nitrogen and phosphorus content, and urease, whereas DSE were more affected by pH.

**Conclusion:**

Drought stress has different effects on AMF and DSE in the roots of *P. euphratica* and *H. ammodendron*. DSE have a greater advantage in extremely arid environments. This study demonstrates the interaction between AMF and DSE with the host plants *P. euphratica* and *H. ammodendron* as well as their effects on the adaptation of host plants to the desert environment, which can provide a basis for strengthening desert vegetation management.

## 1 Introduction

In natural ecosystems, most plants coexist with endophytic fungi, which can effectively promote plant growth and adaptation to stress (Rodriguez et al., 2009). During the long-term succession and evolution of arid desert plants, a series of special strategies for survival in arid environments have developed (Huo et al., 2022). The association between plants and endophytic fungi has also been recognized as a key strategy for desert plants to adapt to stressful environments (Liu et al., 2023), that is, endophytic fungi play an important role in host resistance to drought and other stresses.

Arbuscular mycorrhizal fungi (AMF) are widely distributed soil microorganisms that can form symbioses with a variety of plants (Gai et al., 2006), promote the growth of other soil microorganisms (Sun and Shahrajabian, 2023), transport mineral elements and water to the host plant, and improve the overall stress resistance of plants (Tariq et al., 2023), enabling plants to better survive in desert environments with scarce water and nutrient deficiency (Madouh and Quoreshi, 2023). Therefore, AMF play an important role in desert vegetation and ecological restoration (Alrajhi et al., 2024). As symbionts, dark septate endophytes (DSE) can enhance the absorption of nutrients—such as nitrate, phosphorus, and micronutrients—by plants (Farias et al., 2020; Wang et al., 2023), so that more nutrients are available to the host plants (Mandyam and Jumpponen, 2008). Moreover, the DSE are able to form the “microsclerotia” structure (Madouh and Quoreshi, 2023). Under adverse conditions, DSE increase host plants tolerance to biotic and abiotic stresses by activating physiological and biochemical responses (Ban et al., 2017). Their wide host range, especially its distribution in extreme environments, makes DSE no less ecologically significant than AMF and has potential applications value in ecological conservation and vegetation restoration and more (Malicka et al., 2022). Under natural conditions, AMF and DSE often co-infect plant roots, and their biological characteristics and ecological functions can play a positive role in ecological reconstruction and vegetation restoration after damage (Huo et al., 2021; Xie et al., 2024).

In arid and semiarid zones, changes in the colonization of plant roots by endophytes are mainly caused by changes in soil moisture (Ayala et al., 2023). *P. euphratica* and *H. ammodendron* are, respectively, typical arboreal and shrubby species in oases of arid regions (Tan et al., 2024). *P. euphratica* can regulate climate, prevent sandstorms and desert expansion, and protect oases (Yao et al., 2024). *H. ammodendron* can reduce wind speed, improve forest microclimate, and promote the settlement and growth of other desert plants (Yang and Lv, 2023). Therefore, they play an important role in maintaining the structure and function of the ecosystem. *P. euphratica* and *H. ammodendron* have formed stable colonization structures with AMF and DSE (Dang et al., 2022; Li et al., 2022). Moreover, the two plants have been extensively studied in previous studies, but there is a lack of comprehensive understanding of the colonization patterns of AMF and DSE fungi that colonize simultaneously in the roots of *P. euphratica* and *H. ammodendron*. Based on this, this study considers *P. euphratica* and *H. ammodendron* in the Ebinur Lake Basin as research objects to study the diversity of endophytic fungi, colonization status of the plant root system, and the relationship between colonization status and environmental factors under different moisture conditions. The following questions were raised: (1) What are the differences in the colonization rates and morphological structures of AMF and DSE under different drought conditions? Is there an interaction between these two mycorrhizal fungi, such as competition or mutualistic symbiosis? (2) What are the differences in AMF and DSE species compositions in rhizosphere and roots under different drought conditions? (3) What are the main influencing soil physical and chemical factors causing differences in AMF and DSE colonization across drought gradients?

## 2 Materials and methods

### 2.1 Overview of the study area

Located in Xinjiang Uygur Autonomous Region in northwestern China, Ebinur Lake Wetland National Nature Reserve (EWNR) is the lowest depression and water-salt aggregation center of Ebinur Lake Wetland on the western edge of the Gurbantunggut Desert (44°30′-45°09′N, 82°36′-83°50′E), with a typical desert ecosystem. The annual maximum temperature is 44°C and the minimum temperature is -34°C. The average annual precipitation is less than 100 mm, whereas the annual evaporation is 16 times the average annual precipitation (Li et al., 2023). Ebinur Lake is the largest saltwater lake in Xinjiang. The soils in this basin are mostly grey desert soil, grey brown desert soil, and aeolian sandy soil with severe salinization (Chen et al., 2023). The dominant plant communities are the salt and drought stress tolerant plants such as *P. euphratica*, *H. ammodendron*, and *Tamarix chinensis* (Wang et al., 2022).

### 2.2 Collection of samples

#### 2.2.1 Collection of plant root samples

In May 2023, a sample transect was established north of the Dongdaqiao Management Station within the typical arid desert reserve in the Ebinur Lake Basin, perpendicular to the bank of the Aqikesu River for approximately 500 m, towards the Kumtag Desert. Based on different natural drought conditions, we selected high, medium, and low moisture gradients from near to far at intervals of 1,200 m from the riverbank (representing mild, medium, and severe droughts, respectively), labelled W1, W2, and W3 from high to low moisture. All three sampling points are located in areas with gentle terrain. The main vegetation includes *P. euphratica*, *H. ammodendron* and other herbaceous plants such as *Ceratocarpus arenarius* and *Nitraria tangutorum*. From a high to a low moisture gradient, the height, crown width, and abundance of plants decrease. Three repeated plots were taken for each moisture gradient, and three *P. euphratica* and three *H. ammodendron* plants with similar growth conditions were selected as replicates from east to west in each plot, totaling nine plots, and 54 samples were collected.

Root samples were collected from the concentrated distribution area of fine roots within the 0-20 cm soil layer around the trunk of the tree (Xia et al., 2015), divided, labelled, collected in sterile bags, and immediately sealed and refrigerated. Among the 27 trees, two root samples were taken from each plant for a total of 54 fine root samples, of which 27 root samples were stored at -20°C for the determination of mycelial colonization rate, and the remaining 27 samples were stored at -80°C for DNA extraction. Due to scarce precipitation, local soil water and groundwater are mainly recharged by rivers, and the depth of groundwater decreases with distance from the Aqikesu River, resulting in significant changes in the soil water content gradient (Yang et al., 2022). We measured the water content of the 0-20 cm soil layer in the subsurface. The results showed that in May, the water content of W1, W2, and W3 was 16.54% ± 2.27%, 8.43% ± 1.61%, and 4.12% ± 1.54%, respectively.

#### 2.2.2 Collection of soil samples

The samples were taken to the laboratory, and the surface soil adhering to the roots was shaken off. The root samples were transferred to a sterile 50 ml centrifuge tubes, 20 ml of sterilized 10 m*M* PBS solution was added, the shaker speed was set to 120 rpm, and the centrifuge tubes were placed at room temperature for 20 min. Sterile tweezers were used to remove the root system, the centrifuge speed was set to 6,000 × g, and the remaining suspension was centrifuged at 4°C for 20 minutes. The precipitate obtained after centrifugation was the required rhizosphere soil (He et al., 2021). There were eighteen samples per moisture gradient, for a total of fifty-four soil samples. The shaken soil was then placed in numbered ziplock bags at room temperature.

### 2.3 Determination of soil physical and chemical properties

The soil was collected in an empty aluminum box that had been weighed beforehand, weighed in the field, brought back to the laboratory, dried. The dry soil was weighed to calculate the soil moisture content (SWC). The shaken soil was dried naturally and then sieved for analysis of soil physical and chemical properties, including soil organic carbon (SOC), electrical conductivity (EC), soil pH (pH), total phosphorus (TP), total nitrogen (TN), available phosphorus (AP), ammonium nitrogen (NH_4_-N), nitrate nitrogen (NO_3_-N), urease (UA), and alkaline phosphatase (ALP). The specific methods were as follows (Bao, 2000): organic carbon content was determined by high temperature exothermic potassium dichromate oxidizing capacity method; soil conductivity was measured by precision conductivity meter; pH value was measured by a pH meter in the solution with a water soil ratio of 2.5:1; soil total phosphorus content was determined by HClO_4_-H_2_SO_4_ Mo-Sb colorimetric method; the content of soil total nitrogen was determined by H_2_SO_4_ mixed accelerant digestion; and available nitrogen content was determined by NaHCO_3_ leaching Mo-Sb colorimetric method; KCl leaching-indophenol blue colorimetric method for the determination of soil ammonium nitrogen content; colorimetry method with phenol disulfonic acid for the determination of soil nitrate nitrogen content; phenol-sodium hypochlorite colorimetric method for the determination of soil urease content; and alkaline phosphatase content in soil was determined by phenol-sodium hypochlorite colorimetric method.

### 2.4 Determination of AMF and DSE colonization

Sufficient fine roots were randomly selected from the root samples. The cells were stained using the Phillips and Hayman staining method (Phillips and Hayman, 1970). The structures of AMF and DSE were observed under a microscope (Nikon ECLIPSE Ti2, Japan), and photographs of the arbuscules, hyphae, vesicles of AMF and hyphae, and microsclerotia of DSE were obtained. The root sample observation method and mycorrhizal colonization grading criteria were used to calculate the root colonization rate (F%), colonization density (M%), and arbuscule abundance(A%) (Trouvelot et al., 1986). Microsclerotia colonization rate (MS%) was measured and calculated using the cross method under a 200x microscope (McGONIGLE et al., 1990). F% = number of infected root segments/total number of microscopic examination root segments* 100%. M% = (0.95 * N5 + 0.7 * N4 + 0.3 * N3 + 0.05 * N2 + 0.01 * N1)/total number of root segments examined by microscopy* 100%, where 0.95, 0.7… respectively represent the weight of each level. N5=sum of fifth-level root segments, N4, N3, N2, and N1 have the same meaning. A% = sum of the intersection points of arbuscule colonization/sum of cross colonization points* 100%. MS% = number of cross-colonization points/sum of cross-colonization points * 100%.

### 2.5 Determination of endophytic fungal diversity

Fungal DNA extraction and detection were completed, followed by PCR amplification using ITS1F (CTTGGTCATTTAGAGGAAGTAA) and ITS2R (GCTGCGTTCTTCATCGATGC) primers (Gardes and Bruns, 1993; Lekberg et al., 2018; Wang et al., 2024), and the PCR products were analysed using QuantiFluor ^™^ - ST blue fluorescence quantitative system (Promega Corporation), followed by the construction of an Illumina library and Illumina sequencing. DNA extraction and sequencing were performed by Shanghai Majorbio Bio-Pharm Technology Co., Ltd. (Shanghai, China) to rapidly detect target strains. Then we used UPARSE platform version 7.1 to analyze the obtained gene sequences. To improve the accuracy and stability of the results, we clustered the data sequences and eliminated all chimeric sequences, ensuring that those with 97% similarity were grouped into the same operational taxonomic unit (OTU) to optimize the features of these datasets. An in-depth comparative analysis of the SILVA rRNA database (Release 138 https://www.arb-silva.de/) with the Unite eukaryotic ITS region database (Release 8.0 http://unite.ut.ee/index.php) was performed. The Majorbio cloud computing platform (http://cloud.Majorbio.com) was used to analyze the microbial communities in the soil. The initial data was stored in NCBI Sequence Read Archive database with the accession number PRJNA1046269. The data were initially organized and statistically analysed. By integrating the AMF and DSE taxonomic information and references, we created a species abundance table using the Majorbio platform to screen and download biodiversity information and facilitate subsequent data analysis.

### 2.6 Processing and analysis

SPSS 27 (IBM Corporation, Armonk, NY, USA) was used to perform normality and homogeneity of variance tests on the colonization rates of AMF and DSE, and the colonization rates under different water gradients were compared using multiple group comparisons (Kruskal-Wallis test). Bar charts were plotted using Excel to represent the status of the rhizosphere soil physical and chemical factors in *P. euphratica* and *H. ammodendron*. The ggcor package for R (version 4.3.1) was used to analyze the correlation between fungal colonization in rhizosphere and roots of *P. euphratica* and *H. ammodendron* and soil physical and chemical factors. Heat maps were drawn using the ggpubr, corrplot, and ggplot2 packages to visualize the correlation between the AMF and DSE colonization status. AMF and DSE with different gradients in rhizosphere and roots were classified and visualized by genus using circular and statnet packages for R. Redundancy analysis (RDA) between AMF and DSE colonization status and soil physical and chemical factors in the roots of the two plants was performed using CANOCO 5. Prior to RDA analysis, a species-sample analysis was done using CANOCO 5 to determine the choice of RDA analysis. The collinearity degree between soil physical and chemical factors was assessed by variance inflation factor (VIF) analysis using the R package (version 4.3.0) to ensure that the respective VIF values for all factors were less than 10.

## 3 Results

### 3.1 Soil physical and chemical factors and root AMF and DSE colonization structure characteristics

#### 3.1.1 Analysis of physical and chemical factors in rhizosphere soil

**Figure 1:**
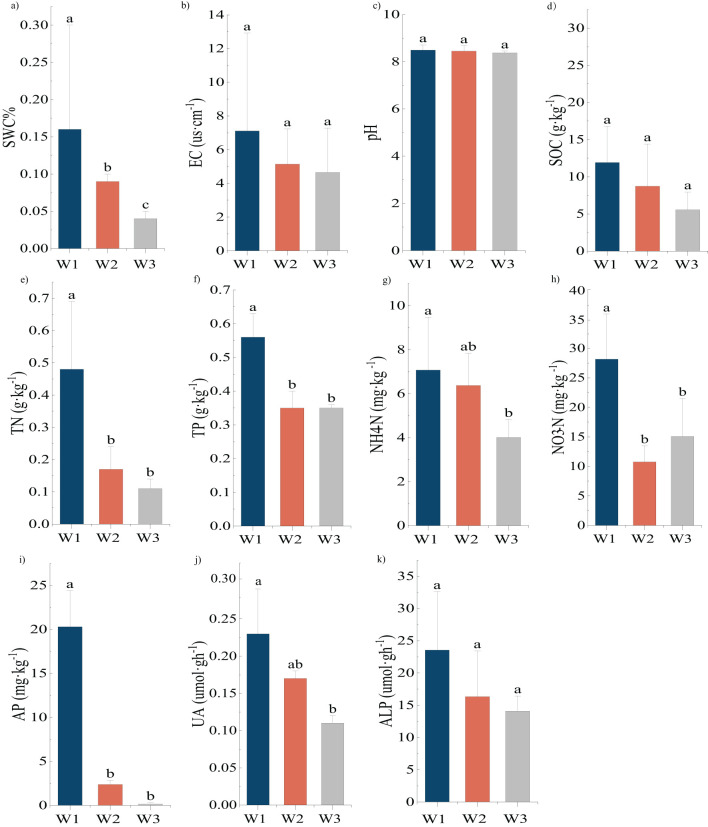
**(A–K)** Differences in physical and chemical properties of soil of *Populus euphratica* (mean ± SD, n=3). Different lowercase letters marked above the bars indicate significant differences in the physical and chemical properties of plant soil (*P*<0.05).

A one-way ANOVA was performed to analyze the significant differences in the physical and chemical properties of the soil of *P. euphratica* (**Figure 1**). SWC decreased with the intensification of drought, and there was a significant difference among the three gradients. Both NH_4_-N and UA decreased with increasing drought severity, and there were significant differences (*P*<0.05) between the contents of the high and low moisture gradients. There was no significant difference between the medium moisture gradient and the other two gradients. The NO_3_-N, content in the high-moisture gradient was significantly higher than those in the medium- and low-moisture gradients (*P*<0.05). EC, pH, SOC, and ALP decreased as the moisture content decreased, but these changes were not significant. TN, TP, and AP decreased with increasing drought severity and the content in the high water gradient were significantly higher than those in the other two gradients (*P*>0.05).

**Figure 2:**
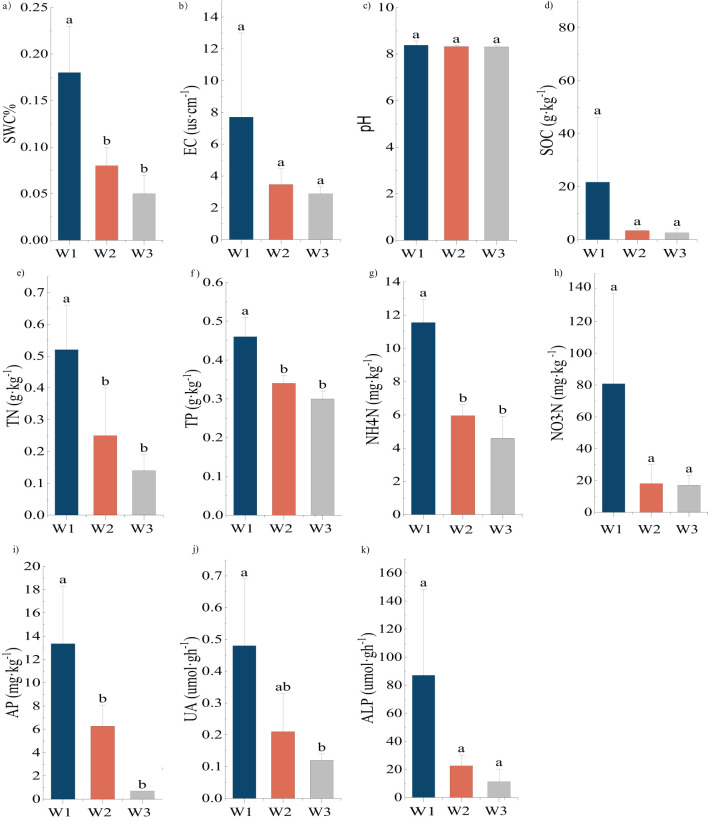
**(A–K)** Differences in physical and chemical properties of soil of *Haloxylon ammodendron* (mean ± SD, n=3). Same as **Figure 1**.

The result of one-way ANOVA in the *H. ammodendron* soil (**Figure 2**) showed that the variation patterns among SWC, TN, TP, NH_4_-N, and AP were the same with significantly greater content under high moisture gradient than in the remaining two gradients (*P*<0.05). There was a significant difference in the UA between the high and low moisture gradients (*P*<0.05), whereas there was no significant difference between the medium moisture gradient and the other two gradients. There were no significant differences in EC, pH, SOC, NO_3_-N, or ALP among the three moisture gradients.

In summary, all physical and chemical factors in soils of the two plants were higher in the high-moisture gradient than in the other two gradients. All physical and chemical factors decreased with a reduction in soil water content, except for NO_3_-N in the rhizosphere soils of *P. euphratica*. TP, TN, and AP were significantly higher (*P*<0.05) in the high moisture gradient than in the medium and low moisture gradients, whereas there were no significant differences between the medium and low moisture gradients.

#### 3.1.2 Colonization characteristics of AMF and DSE fungi

**Figure 3:**
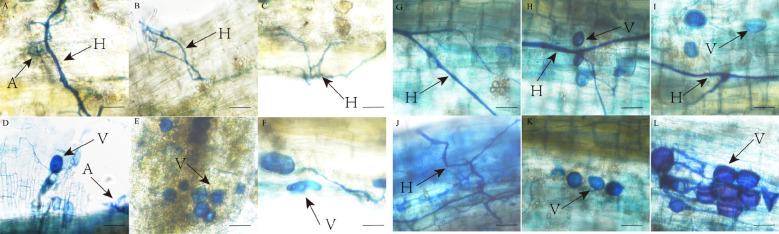
AMF colonization structure in roots of *Populus euphratica* **(A-F)** and *Haloxylon ammodendron* **(G-L)**. A, arbuscule; H, hypha; V, vesicle.

**Figure 4:**
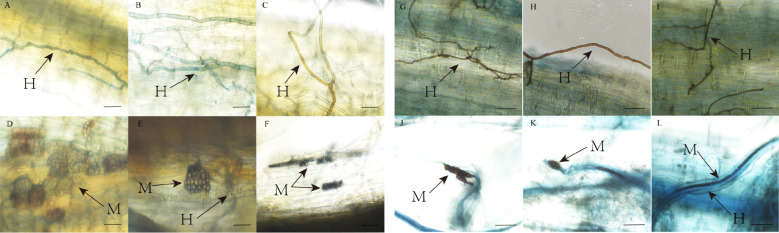
DSE colonization structure in roots of *Populus euphratica* **(A-F)** and *Haloxylon ammodendron* **(G-L)**. H, hypha; M, microsclerotia.

AMF and DSE colonization structures were observed in *P. euphratica* and *H. ammodendron* (**Figures 3**, **4**). AMF and DSE were widely present in the roots of *P*. *euphratica* and *H. ammodendron*. AMF formed typical hyphae, vesicles, and arbuscule. The size and shape of vesicles varied, with both circular and elliptical shapes appearing. DSE formed dark septate hyphae and microsclerotia. The mycelia were mostly brown, and obvious mycelial meshes formed along the longitudinal axis of the roots. Microsclerotia were formed by closely packed and enlarged cells with thickened cell walls, with varying degrees of shape, size, and density. The color of the same microsclerotial cluster was brown with varying shades.

### 3.2 Differences and correlation in colonization rates between AMF and DSE in roots

It can be seen that the AMF colonization rate increased significantly from 13.89% to 71.83% in the roots of *H. ammodendron* from high to low water gradients (**Table 1**). The arbuscule abundance of *P. euphratica* decreased with a decrease in soil moisture, whereas that of *H. ammodendron* increased with a decrease in soil moisture. From high to low water gradients, the DSE colonization rate increased significantly from 53.24% to 92.41% in the roots of *P. euphratica*, and decreased significantly from 67.10% to 45.06% in the roots of *H. ammodendron* (**Table 2**). Similar to *P. euphratica*, the DSE colonization rate and density of *H. ammodendron* in the medium water gradient were the lowest among the three gradients, and the number of microsclerotia was the highest among the three gradients. In most cases, both AMF and DSE had higher colonization rates under low water gradient conditions, indicating that a certain degree of drought promotes the formation of mycorrhizal structures between AMF, DSE and plant roots.

**Table 1:** Differences in AMF colonization rates in the roots of *Populus euphratica* and *Haloxylon ammodendron* under different water gradients.

Endophyte	Category	Species	W1	W2	W3
AMF	F%	*Populus euphratica*	60.15aA	0.00bA	85.42aA
		*Haloxylon ammodendron*	13.89bB	28.42bA	71.83aA
	M%	*Populus euphratica*	9.14abA	0.00bA	21.92aA
		*Haloxylon ammodendron*	0.14bA	0.55bA	22.77aA
	A%	*Populus euphratica*	6.14aA	0.00aA	5.59aA
		*Haloxylon ammodendron*	0.04aA	0.12aA	7.99aA

Different uppercase letters indicate significant differences in the same environmental status between two different plants. Different lowercase letters indicate significant differences in colonization status on water gradients.

**Table 2:** Differences in DSE colonization rates in the roots of *Populus euphratica* and *Haloxylon ammodendron* under different water gradients.

Endophyte	Category	Species	W1	W2	W3
DSE	F%	*Populus euphratica*	54.24bA	48.89bA	92.41aA
		*Haloxylon ammodendron*	67.10aA	23.75cB	45.06bB
	M%	*Populus euphratica*	28.15aA	11.98aA	33.99aA
		*Haloxylon ammodendron*	11.07aA	0.32aA	3.43aB
	MS%	*Populus euphratica*	10.90bA	46.56aA	23.89bA
		*Haloxylon ammodendron*	31.82aA	35.44aA	17.69aA

Same as **Table 2**.

Different uppercase letters indicate significant differences in the same environmental status between two different plants. Different lowercase letters indicate significant differences in colonization status on water gradients.

Under the same water gradient, the colonization status of AMF and DSE in *P. euphratica* and *H. ammodendron* differed. Under a high water gradient, the AMF colonization rate of *P. euphratica* was significantly higher than that of *H. ammodendron*. AMF were not detected in the roots of *P. euphratica* under a medium water gradient, but were detected in all water gradients of *H. ammodendron.* The DSE colonization rate of *P. euphratica* in the medium water gradient was significantly higher than that of *H. ammodendron*. Under a low water gradient, the DSE colonization rate, colonization density, and number of microsclerotia in the roots of *P. euphratica* were significantly higher than in *H. ammodendron*.

**Figure 5:**
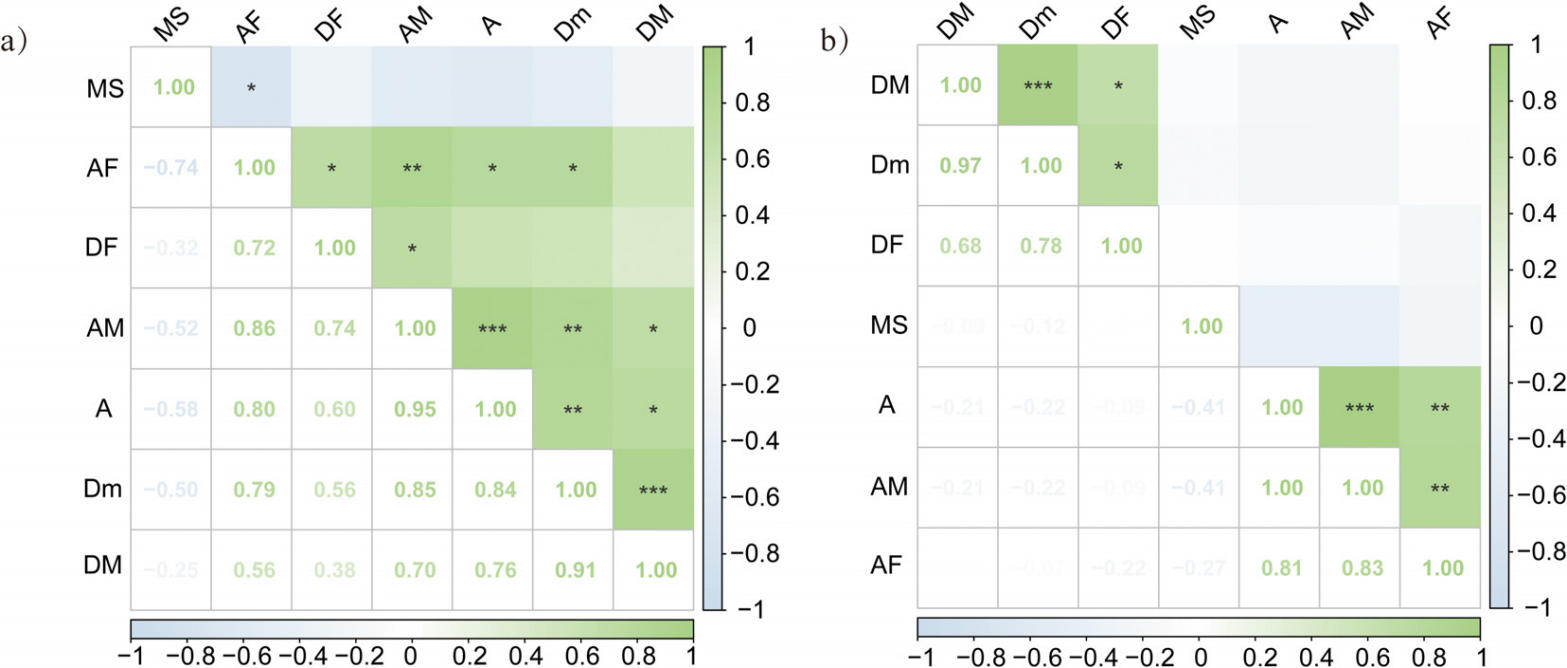
Correlation between DSE and AMF colonization status of **(A)** *Populus euphratica*, **(B)** *Haloxylon ammodendron*. MS, the microsclerotia colonization rate; AF, the colonization rate of AMF; AM, the colonization density of AMF; A, the richness of root hyphae; DF, the colonization rate of DSE; DM, the colonization density of DSE; Dm, the colonization density of DSE in the infected root segment. The asterisk indicates significance p-value. No mark when *P*≥0.05; when 0.01<*P*<0.05, marked * ; when 0.001<*P*<0.01, marked ** ; when *P*≤0.001, marked ***.

Different interactions were observed between DSE and AMF in the roots of *P. euphratica* and *H. ammodendron* (**Figure 5**). For example, in the roots of *P. euphratica*, AMF colonization (AM) was significantly positively correlated with the DSE colonization rate (DF) (R=0.74), and DF was significantly positively correlated with the colonization rate of AMF (AF) roots (R=0.72).

### 3.3 Diversity and abundance differences of AMF and DSE in the root and rhizosphere

**Figure 6:**
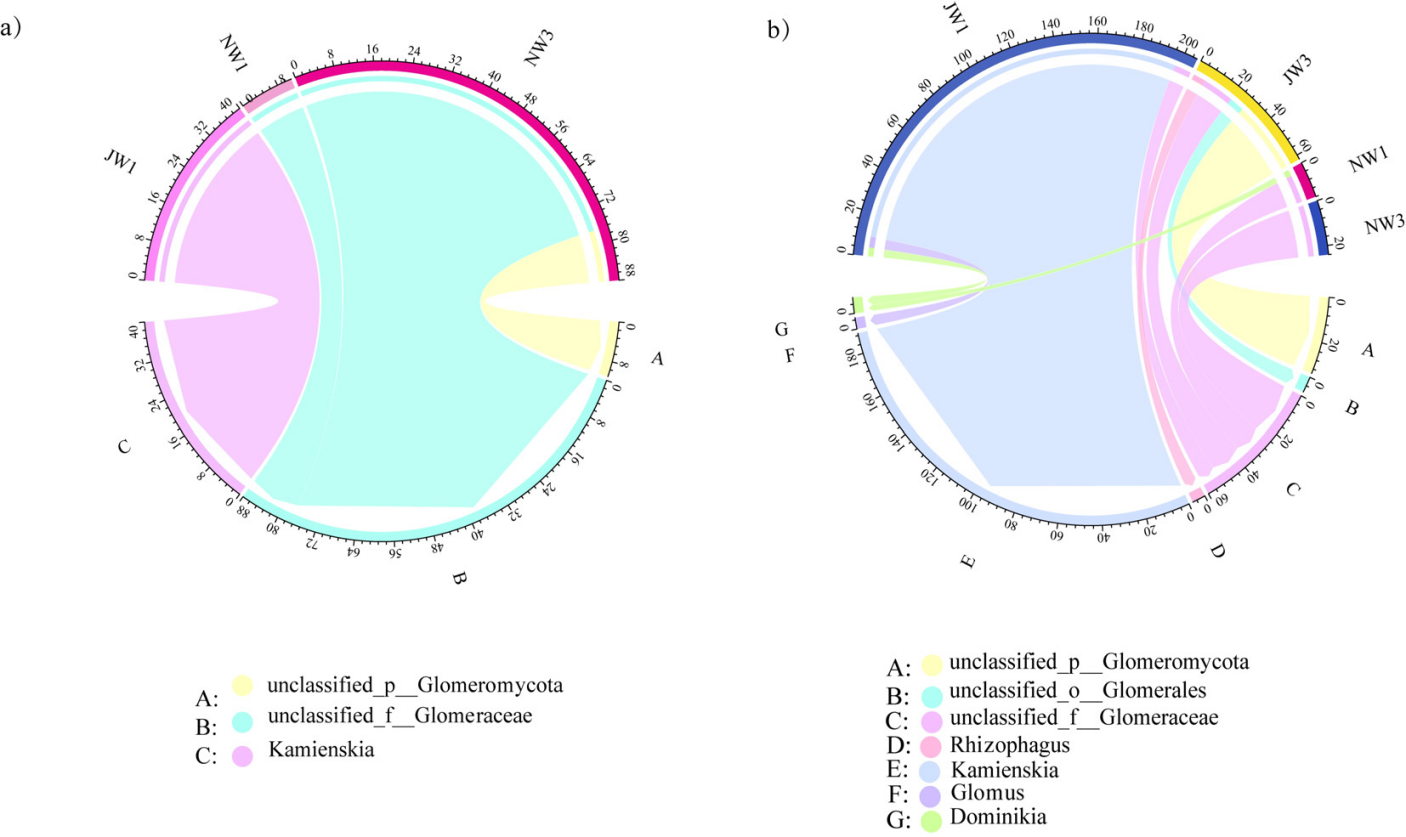
Differences in AMF diversity between *Haloxylon ammodendron* and *Populus euphratica* under different water gradients **(A)** *Haloxylon ammodendron*, **(B)** *Populus euphratica*. J, the sample taken from the rhizosphere; N, the sample taken from the plant roots.

Among all the 27 samples from the rhizosphere soil and roots of *P. euphratica*, 18 samples were detected in seven genera of AMF, with samples belonging to high and low water gradients in the rhizosphere and roots (**Figure 6**). More than one AMF genus coexisted in each of the 18 samples. At low water gradients, the dominant AMF genera from the rhizosphere to the root interior changed from *Kamienskia* to *unclassified_f__glomerraceae*. Under high water gradients, the dominant genus changed from *unclassified_p__Glomeromycota* in the rhizosphere to *unclassified_f__Glomerraceae* in the roots. Overall, *Kamienskia* had the highest abundance, followed by *unclassified_f__Glomerraceae*.

Among the 27 samples in the rhizosphere soil and within the roots of *H. ammodendron*, 18 samples were detected in three genera of AMF, belonging to high water gradients in the rhizosphere and high and low water gradients in the roots, all of which had AMF coexisting in one or more genera. Only two types of AMF were detected in the roots of *H. ammodendron* under a high water gradient, with *unclassified_p__Glomeromycota* being the dominant genus. Under a low water gradient, the dominant genus in the rhizosphere was *Kamienskia*, and the dominant genus in the root was *unclassified_p__Glomeromycota*.

The diversity of AMF species inside and outside the roots of *P. euphratica* was greater than that in *H. ammodendron*. Only three of the seven AMF genera were detected inside and outside the roots of *H. ammodendron*, while *P. euphratica* detected all seven genera including *unclassified_o__Glomerales*, *Rhizophagus*, *Glomus*, and *Dominikia*, with a total number greater than that of *H. ammodendron*. Among all AMF-detected samples, the dominant genus was *unclassified_f__Glomerraceae*. The AMF in the rhizosphere soil of *P. euphratica* was approximately six times the amount of *H. ammodendron*, whereas the amount of AMF in *H. ammodendron* is 2 times more than that of *P. euphratica*.

**Figure 7:**
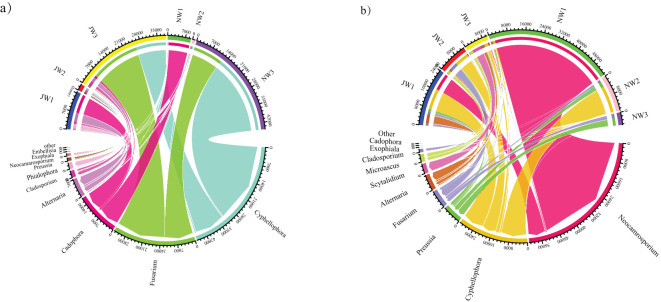
Differences in TOP 10 DSE genera diversity between *Haloxylon ammodendron* and *Populus euphratica* under different water gradients **(A)** *Populus euphratica*; **(B)** *Haloxylon ammodendron*. Same as **Figure 6**. J, the sample taken from the rhizosphere; N, the sample taken from the plant roots.

In total, 25 DSE genera were detected in the rhizosphere soil and roots of *P. euphratica*, and more than one DSE genus coexisted in all 27 samples (**Supplementary Table S2**). The dominant genera were *Cyphellophora*, followed by *Fusarium* (**Figure 7**). The composition of DSE genera of *P. euphratica* varied among the different water gradients. *Cyphellophora* and *Fusarium* have been widely detected in low water gradients. However, *Cadophora*, the dominant genus in the high-water gradients were rarely detected in the low water gradients.

In total, 25 DSE genera were detected in the rhizosphere soil and roots of *H. ammodendron*, and more than one DSE genus coexisted in all 27 samples. The three most abundant genera were *Neocamrosporium*, *Cyphellophora*, and *Preussia*. *Neochamrosporium* was widely distributed in high water gradients in roots, whereas *Scytalidium* was mainly detected in the low water gradient rhizosphere soil. Other genera of DSE were detected in samples from multiple water gradients. The abundance of DSE in the roots of each water gradient was lower than that in the rhizosphere. But the quantities of DSE in the rhizosphere were greater than that in the rhizosphere.

The TOP 10 DSE genera inside and outside the roots of the two plant species were also different: *Scytalidium* and *Microascus* were not dominant in the roots of *P. euphratica*, and *Cadophora, Phialophora*, and *Embellisia* were not dominant in the collected samples of *H. ammodendron* roots.

### 3.4 Effects of soil physical and chemical factors on AMF and DSE colonization

**Figure 8:**
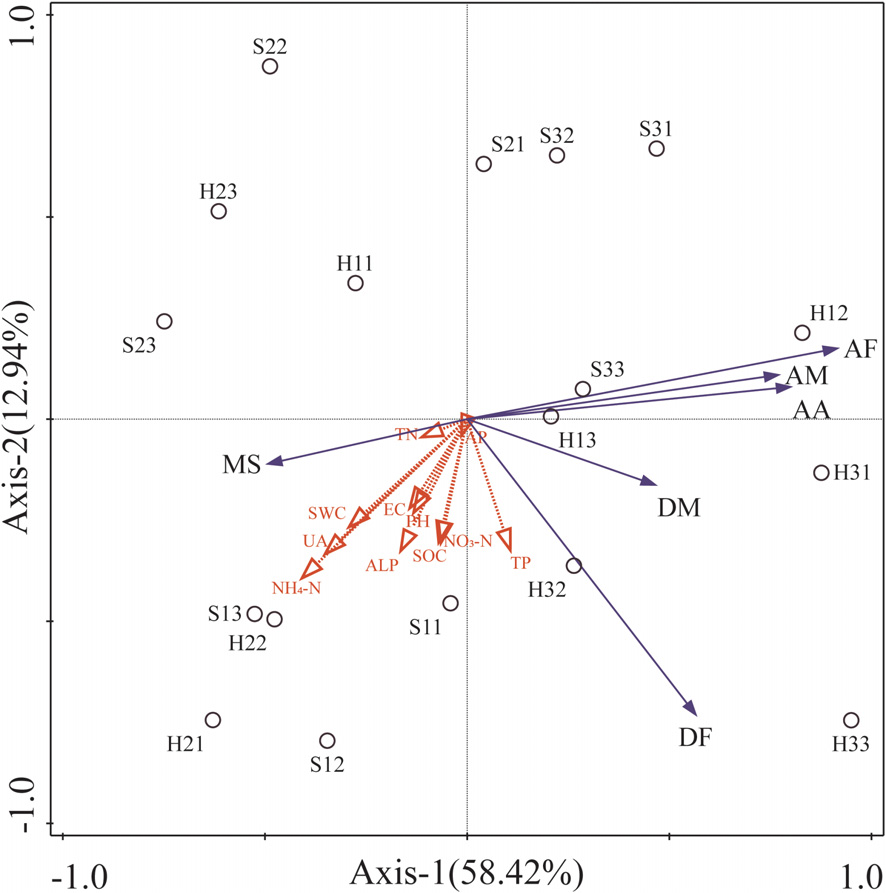
Redundancy analysis of environmental factors and colonization status of *Populus euphratica* and *Haloxylon ammodendron*. AM, the colonization density of AMF; A, the richness of root hyphae; DF, the colonization rate of DSE; DM, the colonization density of DSE; MS, the microsclerotia colonization rate. S, *Haloxylon ammodendron*; H, *Populus euphratica*; the number represents the moisture gradient and sampling number, for example, S11, the No.1 *Haloxylon ammodendron* sample collected at a high moisture gradient.

A redundancy analysis was conducted on the relationship between AMF and DSE colonization status of the roots of *P. euphratica* and *H. ammodendron* and soil physical and chemical factors at the plots. The results showed that the eigenvalues of the first and second sorting axes were 0.5842 and 0.1294, respectively. The two axes explained 71.36% of the changes in endophytic fungi, indicating that the first and second sorting axes could better reflect the changes between endophytic fungi and soil physical and chemical factors (**Figure 8**). The first axis was positively correlated with soil physical and chemical factors TP, negatively correlated with other soil physical and chemical factors, such as pH, AP, NO_3_-N, ALP, UA, SOC, SWC, NH_4_-N, TN and EC. It was positively correlated with AA, AM, AF, DF, and DM and negatively correlated with MS. The second axis was negatively correlated with MS, DF, DM, and all environmental factors, and positively correlated with AM, AF, and AA.

The results indicated (**Supplementary Table S1**) that SWC, AP, TP, and TN were significantly and positively correlated with AM (*P*<0.01). UA levels were significantly positively correlated with AM (*P*<0.01), and significantly negatively correlated with AF (*P*<0.05). ALP levels were significantly positively correlated with AM (*P*<0.01). There was a significant negative correlation between NH4-N and AF (*P*<0.05). There was a highly significant negative correlation between NO3-N and AF (*P*<0.01) and a significant positive correlation with DM (*P*<0.05). PH was significantly and positively correlated with DF (*P*<0.05) and DM (*P*<0.05).

## 4 Discussion

### 4.1 Differences and correlation of colonization rates between AMF and DSE in roots

Soil moisture can directly affect the growth and development of mycorrhizal fungi and the formation of mycorrhizal symbioses (Liu et al., 2018). Under low water gradient, for example, endophytic fungi can more effectively connect the roots of *H. ammodendron* to the surrounding soil microenvironment, thus exhibiting more significant functions in absorbing and transporting water and nutrients. In the present study, the DSE colonization rate of *H. ammodendron* decreased with decreasing water content. We speculate that this may be due to the drought stress exceeding a certain limit, which may have resulted in the production of certain substances by fungal cells (Pegler and Allen, 1993), leading to changes in the cell membrane permeability of *H. ammodendron* (Dick et al., 1996) and affecting the growth of fungi and plants. The colonization rate of AMF in the roots of *P. euphratica* and *H. ammodendron* was negatively correlated with soil water content, consistent with previous findings (Shen and Zhu, 2021). It has been speculated that appropriate level of humidity is beneficial for AMF colonization and spore germination (Frew, 2023). In this study, it was also found that under the same drought stress conditions, host plant species have an impact on the biomass and colonization rate of mycorrhizal fungi (Ilyas et al., 2024). For example, in contrast to the changes in the DSE colonization rate of the roots of *H. ammodendron* mentioned earlier, the DSE colonization rate of *P. euphratica* was negatively correlated with soil moisture content. This may be because the soil moisture content of the W1 plot in this study was more suitable for DSE colonization of the roots of *P. euphratica*. Under these conditions, DSE can have a relatively tight colonization of the roots of *H. ammodendron* (Ge et al., 2018). In our study, we also found that the total colonization rate of DSE under a low water gradient was higher than that of AMF, which not only indicates that DSE has a stronger colonization ability than AMF, but also indicates that DSE has greater colonization advantages under extreme environments (Alberton et al., 2010; Postma et al., 2007). The changes in the colonization structure that occurred in AMF and DSE with increasing drought severity may be an adaptation of endophytic fungi to environmental influences (Xie et al., 2017).

Zhao et al. (2021) found a positive correlation between the colonization rate of DSE microsclerotia and the content of AMF groups, which is consistent with the results of this study. In the present study, we also found a correlation between DSE colonization and AMF in the roots of the two plants. The colonization rate and density of AMF in *P. euphratica* were significantly positively correlated with the colonization rate of DSE, whereas the colonization rate and density of AMF were negatively correlated with the DSE colonization rate and density in *H. ammodendron*. This indicates that AMF and DSE can form different colonization relationships in the roots of different species, and that the interaction between the two mycorrhizal fungi is complex and greatly influenced by the host plant and environmental conditions. This is consistent with previous research results. The study of Shang et al. (2021) study showed that the colonization of AMF and DSE in the roots of cultivated *Vaccinium uliginosum* in Guizhou is highly significantly negatively correlated. Seerangan and Thangavelu, (2014) found that there was no clear relationship between AMF colonization and DSE colonization when studying plants growing in aquatic and wetland habitats, and the co-occurrence of AMF and DSE is more common in terrestrial habitats.

### 4.2 Diversity and abundance differences of AMF and DSE in the root and rhizosphere

In this study, we found that the majority of AMF and DSE were not unique to *P. euphratic*a or *H. ammodendron* but were shared by both, indicating that AMF and DSE strains do not exhibit strong host specificity in arid areas (Liu et al., 2017; Bi and Xie, 2021). For example, *unclassified_f:Glomeraceae*, an unclassified member of the *Glomeraceae* family, was found in the roots of *both H. ammodendron* and *P. euphratica*. *Glomeraceae* is a widely distributed community in the AMF population currently discovered, and was found in both soil and plant roots (Stürmer et al., 2018). Unlike AMF, DSE exhibits host preference (Stroheker et al., 2021). For example, *Cyphellophora*, the dominant genus of DSE, had a significantly greater amount of colonization in the rhizosphere soil and roots of *P. euphratic*a than *H. ammodendron*, whereas another genus, *Neocamrosporium*, had a higher number in the rhizosphere soil and roots of *H. ammodendron*, which also reflects the varying degrees of host preference for different mycorrhizal fungi (Joshee et al., 2009). Under the same drought conditions, there was a significant difference in the colonization status of AMF between the roots and rhizospheres of *P. euphratica*. The diversity and quantity of AMF in the rhizosphere soil were higher than in the roots, which is consistent with the results of Tian et al (2018). on AMF in the rhizosphere of potato roots. Differences in AMF communities between the soil and roots may be determined by the heterogeneity of the rhizosphere soil (Liu et al., 2009). Shi et al. (2022) and Wang et al. (2010) conducted a diversity survey of AMF in the Xinjiang region and found that *Glomus* was the dominant genus. In this study, *Rhizophagus* and *Kamienskia* were more dominant than *Glomus*, which may be due to differences in hosts.

Many of the AMF and DSE genera detected in this study are highly beneficial to the host. For example, *Glomus* genera are used for Se biofortification (Ye et al., 2020). The *Phialocephala* genus can promote the biological adaptability of *Saussurea involucrata* and *Rhodiola rosea* to extremely cold and strong radiation environments (Chen et al., 2018), and improve the gold tolerance properties of host plants. *Thielavia* in the soil provides plants with highly active endoglucanases, increases the accumulation of plant glucose, and promotes the absorption of soil nutrients by plants, thereby increasing the yields of *Medicago sativa* (Medicago sativa Linn) and *Zea mays* (Badhan et al., 2014; Shi et al., 2019). These findings demonstrated that endophytic fungi play an important role in improving plant adaptability to the environment.

### 4.3 Effects of soil physical and chemical factors on the colonization status of AMF and DSE

Studies have shown that the distribution of endophytic fungi that colonize plant roots is related to soil physical and chemical factors (Wang et al., 2015). Both redundancy and correlation analyses in this study indicated that soil physical and chemical factors have different effects on fungal colonization within roots. Nitrogen deposition is one of the most important global change factors (Tang et al., 2023), and nitrogen content plays a role in AMF colonization. When the distribution ratio of nitrogen, phosphorus, and potassium in the soil is unbalanced, the infectivity of AMF is improved, helping plant roots absorb more nutrients from the soil to cope with adversity (Zhou et al., 2021). Increasing nitrogen fertilizer application can promote the proliferation of bacteria and actinomycetes in the rhizosphere soil of *Oryza sativa*, and the nitrogen application level is positively correlated with the number of bacteria and actinomycetes. TP and ALP were highly significantly positively correlated with AMF colonization density, which is consistent with the results of Li et al. (2021). Soil phosphatase can promote the hydrolysis of organic phosphorus compounds into inorganic phosphorus, thereby increasing the available phosphorus content in the soil (Luo et al., 2022). However, under high phosphorus levels, the colonization rate of AMF significantly decreases (Salmeron-Santiago et al., 2023), which is not conducive to plant growth. Therefore, controlling and managing soil phosphorus levels are beneficial for improving the richness and diversity of soil AMF, thereby facilitating the establishment of sustainable ecosystems (Qin et al., 2020). It is obvious that the soil moisture content was not the reason for the lack of AMF colonization in the roots of *P. euphratica* under a moderate water gradient, and the soil moisture content did not cause AMF colonization in the other two gradients. We speculate that this may be due to the aforementioned soil physical and chemical factors (Yan et al., 2023). Under nitrate-nitrogen conditions, endophytic fungal inoculation can promote seedling growth (Sun et al., 2022). Nitrate-nitrogen can reflect soil nitrogen levels, and nitrogen can accelerate the growth metabolism of soil microorganisms (Qu et al., 2022), strongly supporting the results of our study. In this study, there was a significant positive correlation between pH and the colonization status of the DSE communities, which is consistent with previous studies. Hou (2020) found that the colonization rate of DSE was positively correlated with pH in his research on various desert plants. This may be because the pH affects the soluble salt content in the soil and the material exchange capacity of DSE, thus affecting its growth status of DSE (Zhang et al., 2015).

## 5 Conclusion

Our study investigated the effects of soil factors on the colonization of AMF and DSE in the rhizosphere and roots of the desert plants *P. euphratica* and *H. ammodendron* in Xinjiang. This was achieved by observing their colonization status, community composition, morphological characteristics, and differences in morphological colonization structure. The results showed that drought stress increased the colonization rate of DSE in the roots of *P. euphratica* and AMF in the roots of *P. euphratica* and *H. ammodendron* but decreased the colonization rate of DSE in the roots of *H. ammodendron*. The DSE was more dominant in extreme drought environments. The AMF diversity decreased with increasing drought severity, whereas the DSE diversity increased. DSE showed stronger adaptability to drought-stressed environments. The dominant and endemic species of DSE vary in different environments and hosts. AMF were more susceptible to soil factors such as SWC, soil nitrogen and phosphorus content (including AP, TN, TP, NH_4_-N, and NO_3_-N), and UA, whereas DSE was more affected by pH. The results of this study are conducive to the development of targeted and biased protection strategies for desert plants, especially *P. euphratica* and *H. ammodendron*, and provide a basis for endophytic fungal species selection in ecological restoration.

## Data Availability

The datasets presented in this study can be found in online repositories. The names of the repository/repositories and accession number(s) can be found below: https://www.ncbi.nlm.nih.gov/, PRJNA1046269.
