# Supplementary material for: Interaction between arbuscular mycorrhizal fungi and dark septate endophytes in the root systems of Populus euphratica and Haloxylon ammodendron under different drought conditions in Xinjiang, China
Source: Front Plant Sci. 2025 Jan 27;15:1504650. doi: 10.3389/fpls.2024.1504650 (PMC11808033; doi:10.3389/fpls.2024.1504650)
Supplement: Supplementary file 1 [file Table1.docx]

Table S1 The correlation matrix between soil physical and chemical factors and the infection status of AMF and DSE. The green background indicates a significant correlation with infection status (*P*<0.05). Note: Same as Figure 9.

|  | AF | | AM | | AA | | DF | | DM | | MS | |
| --- | --- | --- | --- | --- | --- | --- | --- | --- | --- | --- | --- | --- |
|  | *r* | *P* | *r* | *P* | *r* | *P* | *r* | *P* | *r* | *P* | *r* | *P* |
| SWC | -0.12 | 0.6357 | 0.59 | 0.0098 | -0.30 | 0.2218 | 0.01 | 0.9797 | 0.04 | 0.8681 | -0.18 | 0.4772 |
| EC | 0.23 | 0.3692 | 0.05 | 0.8351 | -0.16 | 0.5224 | 0.14 | 0.5776 | 0.12 | 0.6331 | -0.15 | 0.5517 |
| PH | 0.40 | 0.0992 | 0.41 | 0.0907 | -0.01 | 0.9785 | 0.51 | 0.0296 | 0.54 | 0.0217 | -0.10 | 0.6806 |
| SOC | -0.19 | 0.4494 | -0.12 | 0.6455 | -0.28 | 0.2533 | -0.36 | 0.1411 | -0.39 | 0.1134 | -0.15 | 0.5594 |
| AP | 0.04 | 0.8821 | 0.72 | 0.0007 | -0.09 | 0.7229 | 0.11 | 0.6598 | 0.27 | 0.2868 | -0.26 | 0.2908 |
| TP | -0.26 | 0.2993 | 0.93 | 0.0000 | -0.24 | 0.3399 | 0.11 | 0.6541 | 0.02 | 0.9350 | 0.00 | 0.9886 |
| TN | -0.41 | 0.0928 | 0.88 | 0.0000 | -0.39 | 0.1083 | -0.05 | 0.8573 | -0.19 | 0.4446 | 0.14 | 0.5674 |
| NH4-N | 0.49 | 0.0405 | -0.43 | 0.0779 | 0.02 | 0.9518 | 0.26 | 0.2893 | 0.39 | 0.1104 | -0.07 | 0.7950 |
| NO3-N | 0.70 | 0.0011 | -0.3 | 0.2307 | 0.36 | 0.1445 | 0.29 | 0.2385 | 0.54 | 0.0207 | -0.38 | 0.1216 |
| UA | -0.56 | 0.0159 | 0.68 | 0.0020 | -0.30 | 0.2314 | -0.17 | 0.5095 | -0.41 | 0.0920 | 0.12 | 0.6432 |
| ALP | -0.24 | 0.3311 | 0.93 | 0.0000 | -0.29 | 0.2460 | 0.15 | 0.5566 | 0.01 | 0.9531 | -0.11 | 0.6513 |
