# Supplementary material for: Interaction between arbuscular mycorrhizal fungi and dark septate endophytes in the root systems of Populus euphratica and Haloxylon ammodendron under different drought conditions in Xinjiang, China
Source: Front Plant Sci. 2025 Jan 27;15:1504650. doi: 10.3389/fpls.2024.1504650 (PMC11808033; doi:10.3389/fpls.2024.1504650)
Supplement: Supplementary file 2 [file Table2.docx]

Table S2 Dominant genera of DSE and references for identifying them as DSE

| Genus | Reference |
| --- | --- |
| *Alternaria* | (He et al., 2021) |
| *Cadophora* | (Maciá-Vicente et al., 2020) |
| *Cladosporium* | (Vohnik, 2022) |
| *Cyphellophora* | (Yao et al., 2019) |
| *Exophiala* | (Yuan et al., 2010) |
| *Embellisia* | (Li et al., 2018) |
| *Fusarium* | (Wilberforce et al., 2003) |
| *Microascus* | (He et al., 2022) |
| *Neocamarosporium* | (He et al., 2022) |
| *Phialophora* | (Xu et al., 2015) |
| *Preussia* | (He et al., 2021) |
| *Scytalidium* | (He et al., 2020) |

Note: The references are only for the purpose of confirming that these genera belong to DSE and have no other significance in this manuscript

Reference

Ge J., 2019. Ecological heterogeneity of DSE fungus in Anxi extreme arid desert environment in China (Master’s thesis). Hbei University.

He, C., Han, T., Tan, L., Li, X., 2022. Effects of Dark Septate Endophytes on the Performance and Soil Microbia of Lycium ruthenicum Under Drought Stress. Front. Plant Sci. 13. https://doi.org/10.3389/fpls.2022.898378

He, C., Wang, W., Hou, J., 2020. Plant performance of enhancing licorice with dual inoculating dark septate endophytes and Trichoderma viride mediated via effects on root development. BMC Plant Biology 20, 325. https://doi.org/10.1186/s12870-020-02535-9

He, C., Wang, W., Hou, J., Li, X., 2021. Dark Septate Endophytes Isolated From Wild Licorice Roots Grown in the Desert Regions of Northwest China Enhance the Growth of Host Plants Under Water Deficit Stress. Front. Microbiol. 12. https://doi.org/10.3389/fmicb.2021.522449

Hu L., Yan S., Sun M., Li W., Gu P., 2019. Infection characteristics and diversity of dark septate endophytic fungi isolated from Lycium barbarum in Ningxia. Microbiology China 46, 2973–2984. https://doi.org/10.13344/j.microbiol.china.180903

Hu Q., 2023. Species Diversity and Drought Tolerance of Dark Septate Endophytes Fungi in Desert Plants (Master’s thesis). Hebei University. https://doi.org/10.27103/d.cnki.ghebu.2022.002174

Li, X., He, X., Hou, L., Ren, Y., Wang, S., Su, F., 2018. Dark septate endophytes isolated from a xerophyte plant promote the growth of Ammopiptanthus mongolicus under drought condition. Sci Rep 8, 7896. https://doi.org/10.1038/s41598-018-26183-0

Lin H., 2022. Study on Dark Septate Endophytic fungi (DSE) and soil nutrients of Ulmus pumila L. in sandy land in eastern Inner Mongolia. (Master’s thesis). Inner Mongolia Agricultural University. https://doi.org/10.27229/d.cnki.gnmnu.2022.001351

Maciá-Vicente, J.G., Piepenbring, M., Koukol, O., 2020. Brassicaceous roots as an unexpected diversity hot-spot of helotialean endophytes. IMA Fungus 11, 16. https://doi.org/10.1186/s43008-020-00036-w

Vohnik, M., 2022. Are lulworthioid fungi dark septate endophytes of the dominant Mediterranean seagrass *Posidonia oceanica?* Plant Biol. 24, 127–133. https://doi.org/10.1111/plb.13353

Wilberforce, E.M., Boddy, L., Griffiths, R., Griffith, G.W., 2003. Agricultural management affects communities of culturable root-endophytic fungi in temperate grasslands. Soil Biology and Biochemistry 35, 1143–1154. https://doi.org/10.1016/S0038-0717(03)00176-7

Xu, R., Li, T., Cui, H., Wang, J., Yu, X., Ding, Y., Wang, C., Yang, Z., Zhao, Z., 2015. Diversity and characterization of Cd-tolerant dark septate endophytes (DSEs) associated with the roots of Nepal alder (*Alnus nepalensis*) in a metal mine tailing of southwest China. Appl. Soil Ecol. 93, 11–18. https://doi.org/10.1016/j.apsoil.2015.03.013

Yao, Q., Xu, Y., Liu, X., Liu, J., Huang, X., Yang, W., Yang, Z., Lan, L., Zhou, J., Wang, G., 2019. Dynamics of soil properties and fungal community structure in continuous-cropped alfalfa fields in Northeast China. PeerJ 7, e7127. https://doi.org/10.7717/peerj.7127

Yuan, Z., Zhang, C., Lin, F., Kubicek, C.P., 2010. Identity, Diversity, and Molecular Phylogeny of the Endophytic Mycobiota in the Roots of Rare Wild Rice (*Oryza granulate*) from a Nature Reserve in Yunnan, China. Appl. Environ. Microbiol. 76, 1642–1652. https://doi.org/10.1128/AEM.01911-09
